# Health Literacy 2.0: Integrating Patient Health Literacy Screening with Universal Precautions

**DOI:** 10.3928/24748307-20191028-02

**Published:** 2019-12-05

**Authors:** Kristie B. Hadden, Sunil Kripalani

The idea of screening patients for low health literacy has had a polarizing effect in the health literacy community. One side feels that universal precautions and screening for low health literacy are mutually exclusive; meaning, if you screen patients for low health literacy, you are not adhering to the universal precautions approach. Others may be concerned about the feasibility of fully scaling universal precautions across a clinical enterprise and favor a more targeted approach that identifies patients with risk factors for low health literacy so that interventions that rely on limited resources can be allocated where there is greatest potential for benefit. Given recent changes in health care delivery models, we propose that the time has come to consider a hybrid approach that employs both the foundation of universal precautions for all patients, as well as identification of those for whom universal precautions alone may not suffice, due to extreme needs. When this combined approach is used, the limitations of each approach are mitigated by the other. This integrated approach is well aligned with recent innovation in the health care landscape and should be considered by researchers, providers, and policymakers.

## Health Literacy Universal Precautions

Health literacy universal precautions were first operationalized in 2010 to address the complex demands faced by patients in health systems in the United States (DeWalt et al., 2011). The approach calls for all health care organizations and professionals to assume that all patients may have difficulty comprehending health information and accessing services (Brega et al., 2015). A well-developed Universal Precautions Toolkit (Brega et al., 2015) provides guidance for practices to conduct a baseline organizational assessment, develop a plan for addressing health literacy, take steps to improve spoken communication (e.g., regular use of Teach Back), improve written patient education materials and signage, and enhance patient empowerment and self-management (e.g., medication management and support systems). Many experts have endorsed this approach that aims to provide a broad safety net for all patients, including those whose challenges may be hidden. These universal precautions also accommodate issues related to the variability of understanding information depending on the context (Paasche-Orlow, Schillinger, Greene, & Wagner, 2006) (i.e., even patients who typically do not struggle with information may experience challenges due to situational factors such as the emotional stress of a new diagnosis or when the condition is rare, serious, or complex).

Although health literacy universal precautions have been broadly supported by researchers, policymakers, and promoters of health literacy, recent publications have revealed barriers to their adoption in health systems. Indeed, some of the patient-level universal precautions (e.g., Teach Back) may be challenging to integrate routinely into busy clinical environments, whereas others (e.g., medication management support) are resource-intensive. Weiss (2017) posits that “Universal precautions are not yet universal,” commenting on Liang and Brach's findings that in 2017 only 70% of patients reported always receiving instructions that are easy to understand and 29% reported the use of Teach Back to confirm understanding (Liang & Brach, 2017). Although the study highlighted some encouraging progress, health literacy universal precautions were described as “a distant dream” (Liang & Brach, 2017). For health literacy universal precautions to be achieved, these researchers highlight the need to improve providers' health literacy skills and to redesign workflows to integrate health literate practices. Much like the problem of health literacy, it appears that the demands of implementing universal precautions with all patients in our health systems outweigh the capacity of our resources to do so.

## Health Literacy Screening

Researchers have developed efficient methods of screening patients for low health literacy using validated questions that assess patients' self-reported understanding of medical information and forms (Chew, Bradley, & Boyko, 2004; Morris, Maclean, Chew, & Littenberg, 2006). The questions are easy to administer and are acceptable to patients (Cawthon, Mion, Willens, Roumie, & Kripalani, 2014; Heinrich, 2012; Komenaka et al., 2014). Research has shown that 90% of patients feel it is useful for physicians and nurses to know if they are having difficulties related to low health literacy (Farrell, Chandran, & Gramling, 2008; Ryan et al., 2008; Seligman et al., 2005). One way to do this is to incorporate patient health literacy information into health care information systems, which has been recommended by the National Academy of Medicine (Kindig, Panzer, & Nielsen-Bohlman, 2004). This approach was successfully implemented at Vanderbilt University Medical Center in 2010 (Cawthon et al., 2014) as part of the nursing intake process performed by medical assistants and nurses. The University of Arkansas for Medical Sciences (UAMS) used Vanderbilt's implementation model and has been screening patients since 2016. Both institutions use screening items, originally developed and validated by Dr. Lisa Chew (Chew et al., 2004), which we refer to as the Brief Health Literacy Screen (BHLS); Vanderbilt uses all three and UAMS uses one question. The items are incorporated into the electronic health record (EHR) as part of the educational needs assessment and usually require less than 1 minute to administer. To date, health literacy screening data have been recorded on more than 615,000 patients across the two health systems in both inpatient and outpatient settings, along with other information related to their educational needs. Although, as with most assessments, the possibility of measurement error exists (Goggins, Wallston, Mion, Cawthon, & Kripalani, 2016), a growing research base demonstrates the construct and predictive validity of the BHLS collected in clinical practice (Boyle et al., 2017; McNaughton et al., 2014; McNaughton et al., 2015; Scarpato et al., 2016; Wallston et al., 2014; Wright et al., 2018). Other examples now exist of screening health literacy either routinely or selectively (Rymer et al., 2018; Sand-Jecklin, Daniels, & Lucke-Wold, 2017).

Patient-level health literacy data make it possible for clinical staff who have completed training in clear health communication to provide additional assistance to patients with low health literacy levels. At a population level, these screening data are also useful for examining the association of health literacy with processes and outcomes of care (Boyle et al., 2017; McNaughton et al., 2014; McNaughton et al., 2015; Scarpato et al., 2016; Wright et al., 2018); for determining whether interventions have a different level of effectiveness by level of health literacy (Yiadom et al., 2018); and for refining risk stratification algorithms. The data are also helpful to raise awareness of the prevalence of low health literacy, both in general and in specific clinical areas, and for developing interventions to improve patient management and outcomes.

Throughout the last decade, concerns have arisen regarding the risk of stigmatizing and embarrassing patients through health literacy screening (Paasche-Orlow & Wolf, 2008). In addition to noting the potential for this harm, Paasche-Orlow and Wolf (2008) also highlighted the limitations of the predictive power of current screening questions and the lack of evidence-based approaches to address the needs for patients who are identified as having limited health literacy in clinical practice. Seligman (2005) published a study that noted no benefit in terms of self-efficacy improvement related to health literacy screening for patients with diabetes, but reported that physicians who were notified of their patients' screening results were “more likely to use management strategies recommended to improve communication with [these] patients” (p. 1005).

Other studies have shown that patients feel that it is useful for their providers to know about their health literacy challenges (Farrell et al., 2008; Ryan et al., 2008; Seligman et al., 2005), and when screening is done systematically and respectfully, patients do not object to clinical health literacy screening (Heinrich, 2012; Komenaka et al., 2014). The studies that demonstrated embarrassment and potential for stigmatization used a “test-like” assessment, rather than self-perception screening questions (Chew et al., 2004) that fit in logically with other screening questions in routine clinical workflows and have been validated for use clinically (Wallston et al., 2014). As with other types of screening, adequate training on administering screening questions is essential to appropriate implementation to ensure valid results and minimize the potential for stigmatization.

## Combining Health Literacy Universal Precautions with Screening

“Health literacy 2.0,” a hybrid approach to addressing health literacy in health systems, integrates universal precautions with targeted assistance for patients with lower health literacy levels. This approach supports striving for clear communication with all patients but recognizes that resources are limited and gaps in implementation of universal precautions are prevalent. Thus, the approach also involves screening to identify patients at greatest risk, so that resources can be directed to support their care.

System-wide screening and documentation in EHRs produces data that can be used for targeted interventions, population health opportunities, and point-of-care best practices that are data-driven, rather than universal or result from profiling. Further, screening patients for low health literacy is in alignment with risk-based models of medical care and models of precision medicine that are prevalent in the current health care landscape and include attention to social determinants of health (Adler & Stead, 2015; Ziegelstein, 2015).

## Health Care Innovation Landscape

“Health literacy 2.0” should acknowledge the limitations of past and current attempts to address health literacy on large scales, learn from research and evidence, and embrace the landscape of innovation that is the context of our current health systems. The hybrid approach of universal precautions and screening for health literacy holds promise in this future. As health care shifts toward more tailored approaches to care, additional evidence is needed about how to best identify and deliver personalized care to patients with risk factors for poor outcomes related to health literacy.

Historically, conceptual and analytical models of health literacy reveal causal pathways that position health literacy as an influencer of health behaviors that determine health outcomes (Paasche-Orlow & Wolf, 2007). Most health care innovations like personalized medicine and Learning Health Systems have a focus on outcomes, data, and tailoring care. Empirical research has demonstrated that not only are many clinical outcomes associated with health literacy, interventions can be effective at improving outcomes, specifically for patients with low health literacy (Berkman et al., 2011; Fernandez-Gutierrez, Bas-Sarmiento, Albar-Marin, Paloma-Castro, & Romero-Sanchez, 2018; Zoellner et al., 2016). Because health literacy is often a modifier of treatment effect, health literacy data can be used to tailor interventions for personalized medicine; meaning, some interventions are more effective for patients with low health literacy and can be tailored to increase the likelihood of success.

A notable health literacy innovation that has influenced how care is and should be delivered in the U.S. is the Ten Attributes of Health Literate Care Organizations model (Brach et al., 2012; Koh, Brach, Harris, & Parchman, 2013). This model has traditionally been viewed through a health literacy universal precautions lens. However, several of the attributes are also well-aligned with personalized, data-driven strategies leveraged by health literacy screening **(Table 1**).

One of the most influential innovations in the health care landscape in the U.S. is the adoption of the Institute for Healthcare Improvement's Triple Aim (2018), which focuses on better care, better health of populations, and lower costs. Patient health literacy data can be used in pursuit of all three of these aims. Patient health literacy data in EHRs can be viewed in real-time so that point-of-care interventions can be implemented to improve the patient experience. Patients with risk factors for poor outcomes due to low health literacy can likewise be identified through EHR reports so that tailored instructions, education, and outreach support can be deployed for their specific conditions and needs to improve outcomes. Finally, by focusing resources on those patients whose needs are the greatest and who are most likely to benefit from health literacy interventions, health care costs can be positively affected.

Other health care delivery model innovations have created a space for health literacy. Health systems, especially those that are a part of an Accountable Care Organization (https://innovation.cms.gov/initiatives/ACO/), can use health literacy “risk” data to identify patients for whom an evidence-based intervention and/or best practices will likely lead to improvement of specific health outcomes. As health systems personalize medicine and medical care, patients' individual capacity to understand information and choices becomes essential. Health literacy screening in patient EHRs can provide data that can also be used in population health strategies, quality and satisfaction efforts, and a host of medical informatics initiatives. There are vast opportunities for health systems researchers and administrators to test and disseminate novel ways of integrating health literacy in this new and evolving landscape.

## Conclusion

In the last 10 years, as health literacy universal precautions have been promoted in the U.S., our health systems have experienced unprecedented change. The integration of EHRs, alternative payment models, personalized medicine, the Triple Aim (Institute for Healthcare Improvement, 2018)/ Quadruple Aim (Feeley, 2017), value-based care, patient and family centered care, medical informatics, population health, and many other innovations have changed the quality, delivery, and reimbursement of medical care dramatically. As the U.S. embraces the changes in “Health 2.0” (Subaiya, 2016) in which patients are empowered to have greater control over their own health, there are robust opportunities to leverage what we know about health literacy in this new context.

Both health literacy universal precautions and a universal screening approach have limitations. Implementation of all of the precautions with all patients is often not feasible in real-world medical settings, given shifting and shrinking resources in our health systems. If some elements of universal precautions (e.g., staff training in use of plain language and Teach Back) are applied as a safety net for all patients, and screening is used to identify patients at greater risk, we can better serve both population and individual patient needs. Screening patients for low health literacy requires a modest amount of staff training to normalize the questions and ask them respectfully, as well as time to collect data (Cawthon et al., 2014). However, once patients are identified, evidence-based and best practices for health literacy can be used either at the point of care or in follow-up to optimize outcomes for those who need it the most. This approach has value in both inpatient and outpatient settings where patients are required to learn new health information and self-management skills. Such efforts could be integrated into broader efforts to measure and address social determinants of health (Adler & Stead, 2015). The decision about which staff perform the screening will likely vary according to institutional staffing models and workflows, but are likely to include medical assistants, technicians, or nurses who perform patient intake assessments. Similarly, the health care professionals who provide additional assistance would vary according to what is being offered (e.g., nurse for disease information, pharmacist for medication counseling). The call to action is not to “do better” with universal precautions, or to “scrap” these precautions and replace them with screening, but to integrate these two approaches to reap more robust benefit.

Although integrating these two approaches may provide opportunity to better serve patients and health systems, the need for evidence-based interventions is significant. As researchers and practitioners respond to the call to innovate, we must ensure that we continue to develop, test, and disseminate new research on interventions that are effective in this new context.

## Figures and Tables

**Table 1:** Alignment of the 10 Attributes of a Health Literate Health Care Organization with Universal and Personalized Approaches

**No.**	**Attribute**	**Universal**	**Personalized**
1	Has leadership that makes health literacy integral to its mission, structure, and operations	X	
2	Integrates health literacy into planning, evaluation measures, patient safety, and quality improvement	X	X
3	Prepares the workforce to be health literate and monitors progress	X	
4	Includes populations served in the design, implementation, and evaluation of health information and services	X	X
5	Meets the needs of populations with a range of health literacy skills while avoiding stigmatization	X	X
6	Uses health literacy strategies in interpersonal communications and confirms understanding at all points of contact	X	
7	Provides easy access to health information and services and navigation assistance	X	
8	Designs and distributes print, audiovisual, and social media content that is easy to understand and act on	X	
9	Addresses health literacy in high-risk situations, including care transitions and communications about medicines	X	X
10	Communicates clearly what health plans cover and what people will have to pay for services	X	

Adapted from Ten Attributes of Health Literate Health Care Organizations by C. Brach, et al, 2012, Institute of Medicine.
